# Clinical and Microbiological Evaluation of a Chlorhexidine-Modified Glass Ionomer Cement (GIC-CHX) Restoration Placed Using the Atraumatic Restorative Treatment (ART) Technique

**DOI:** 10.3390/ma15145044

**Published:** 2022-07-20

**Authors:** Jithendra Ratnayake, Arthi Veerasamy, Hassan Ahmed, David Coburn, Carolina Loch, Andrew R. Gray, Karl M. Lyons, Nicholas C. K. Heng, Richard D. Cannon, Marcus Leung, Paul A. Brunton

**Affiliations:** 1 Sir John Walsh Research Institute, Faculty of Dentistry, University of Otago, P.O. Box 56, Dunedin 9054, New Zealand; arthi.senthilkumar@otago.ac.nz (A.V.); hassan400@yahoo.com (H.A.); david.coburn@otago.ac.nz (D.C.); carolina.loch@otago.ac.nz (C.L.); karl.lyons@otago.ac.nz (K.M.L.); nicholas.heng@otago.ac.nz (N.C.K.H.); richard.cannon@otago.ac.nz (R.D.C.); leung.marcus86@gmail.com (M.L.); 2 Biostatistics Centre, Division of Health Sciences, University of Otago, P.O. Box 56, Dunedin 9054, New Zealand; andrew.gray@otago.ac.nz; 3 Curtin University, Bentley, WA 6102, Australia; paul.brunton@otago.ac.nz

**Keywords:** root caries, atraumatic restorative treatment (ART) technique, glass ionomer cement (GIC), chlorhexidine digluconate (CHX), restoration survival

## Abstract

The aims of this study were to investigate the clinical effectiveness and patient acceptability of a modified glass ionomer cement placed using the atraumatic restorative treatment (ART) technique to treat root caries, and to carry out microbiological analysis of the restored sites. Two clinically visible root surface carious lesions per participant were restored using ART. One was restored with commercial glass ionomer cement (GIC) (ChemFil^®^ Superior, DENTSPLY, Konstonz, Germany) which acted as the control. The other carious root lesion was restored with the same GIC modified with 5% chlorhexidine digluconate (GIC-CHX; test). Patient acceptability and restoration survival rate were evaluated at baseline and after 6 months. Plaque and saliva samples around the test and control restorations were collected, and microbiological analysis for selected bacterial and fungal viability were completed at baseline, and after 1, 3, and 6 months. In total, 52 restorations were placed using GIC and GIC-CHX in 26 participants; 1 patient was lost to follow-up. After reviewing the restorations during their baseline appointments, participants indicated that they were satisfied with the appearance of the restorations (*n* = 25, 96%) and did not feel anxious during the procedure (*n* = 24, 92%). Forty-eight percent (*n* = 12) of the GIC-CHX restorations were continuous with the existing anatomic form as opposed to six for the GIC restorations (24%), a difference which was statistically significant (*p* = 0.036). There was no statistically significant reduction in the mean count of the tested microorganisms in plaque samples for either type of restorations after 1, 3, or 6 months. Restoration of carious root surfaces with GIC-CHX resulted in higher survival rates than the control GIC. ART using GIC-CHX may therefore be a viable approach for use in outreach dental services to restore root surface carious lesions where dental services are not readily available, and for older people and special needs groups.

## 1. Introduction

With the increase in life expectancy, people are retaining more of their natural dentition for longer. As people age, medical conditions and other physical, intellectual and cognitive disabilities can negatively-impact their oral health and affect their overall wellbeing, which places an additional burden on the oral healthcare system.

Dental caries is a global disease affecting all ages and sectors of the population [1,2]. It is a multifactorial disease that occurs as a result of interactions between the host, the environment, and microorganisms. Dental caries can be either an active or an arrested disease process. Active caries usually requires a susceptible host with suitable environmental factors created by high intake of dietary carbohydrates coupled with poor oral hygiene [1,3,4]. Root caries is any carious lesion which occurs on the root surface of the tooth and is caused by the acid produced by bacteria [5]. Older people are at higher risk of developing root caries due to gingival recession exposing the root surface, reduced salivary flow, inadequate oral hygiene, and dietary factors [5]. Reduced salivary flow is often a result of polypharmacy, which is common in older people [6]. Saliva plays a key role in oral lubrication and acid neutralisation [7]. Moreover, older people are more likely to have impaired dexterity and mobility; live in rest homes with medical conditions so that they are more dependent on care; and have poor oral hygiene practices compared with the general population [8,9]. Such a cohort would benefit from a quick and simple method of delivering cost effective root caries treatment that is less stressful than conventional treatment. If appropriate preventative measures are not implemented properly, the risk of developing root caries remains higher for older people. Preventive measures such as fluoride application can reduce the incidence of root caries [10]. Minimal intervention dentistry has gained popularity in recent years, as exemplified by the effective Hall technique and atraumatic restorative treatment (ART) approaches [11,12]. ART can be applied in both the deciduous and permanent dentitions, and has significant advantages compared to conventional restorative techniques. These include being pain-free, not requiring anaesthesia, involving minimal cavity preparation, being cost-effective, and having high restoration survival rates [13,14,15].

The development of wear-resistant glass ionomer cements (GICs) in the mid-1990s replaced medium viscosity glass ionomers, and presently, wear-resistant glass ionomer cements are the material of choice when using ART [16]. Although the development of ART was intended mainly for underprivileged children in developing countries [16], it has gained popularity in the treatment of older people, specifically for root caries.

The antimicrobial properties of restorative materials are important for the long-term success of restorations. These benefits include the prevention of caries recurrence around the margins of restorations, the inhibition of plaque accumulation near restorations, and reduction in the number of cariogenic microorganisms in the salivary fluids and oral cavity [17]. Several studies have evaluated the effect of incorporating antimicrobial agents such as chlorhexidine (CHX) into GIC [17,18,19,20]. CHX has been shown to have great substantivity with human dentine. Furthermore, both the gel and solution forms of CHX have been shown to have up to 90 days of retention in dentine [21]. This study aimed to evaluate the restoration integrity, survival rate, patient acceptability, and antimicrobial performance of chlorhexidine-modified GIC (GIC-CHX) in the treatment of root caries.

## 2. Materials and Methods

### 2.1. Participant Recruitment

Ethical approval was obtained from the Health and Disability Ethics Committee (approval number 16/CEN/174) and the study was conducted in full accordance with the World Medical Declaration of Helsinki: ethical principles for medical research involving human subjects. Participants with two root surface carious lesions that required operative intervention were recruited from the Faculty of Dentistry, University of Otago, New Zealand. The clinical trial was registered in the Australia New Zealand Clinical Trials Registry (https://www.anzctr.org.au accessed 25 May 2022; registration ID ACTRN12616001651471). Participants aged 18 years and over, and who had two or more teeth with root caries, were included in the study. Exclusion criteria included: participants with full dentures, those undergoing current antibiotic therapy, or who have had radiotherapy of the head and neck region in the last 12 months. After written informed consent was obtained, eligible participants received a full oral health assessment and detailed oral instructions were given (i.e., to avoid the use of mouthwashes which could affect the oral microbiota).

### 2.2. Preparation for, and Application of, GIC/GIC-CHX Restorations Using ART

Preparation of the root surface carious lesions was conducted by an experienced dentist (aided by a chairside assistant) according to the World Health Organization ART guidelines [22]. Briefly, the tooth was isolated, plaque was removed from the tooth surface with a wet cotton wool pellet, and the outer carious dentine was removed with excavators. Any unsupported thin enamel/cementum was removed. The cavity was then cleaned with water and dried using dry cotton wool pellets to ensure no plaque or debris was present. The dentine was conditioned using 10% polyacrylic acid (GC Dentin Conditioner™, GC Corporation, Tokyo, Japan) for 20 s. The cavity was then washed again and gently dried with cotton wool pellets. One root caries lesion (on the test tooth) was restored with GIC (ChemFil Superior, DENTSPLY, Konstonz, Germany) mixed with 5% (*v*/*v*) chlorhexidine digluconate (DENTSPLY, Konstonz, Germany) (GIC-CHX)). Glass ionomer cements (GIC) are normally prepared by mixing the GIC powder with deionised or distilled water to form a cement, as per the manufacturer’s instructions. In this study, the water was replaced with an aqueous solution of 5% chlorhexidine digluconate (CHX) to prepare the modified GIC (GIC-CHX).

**Figure 1:**
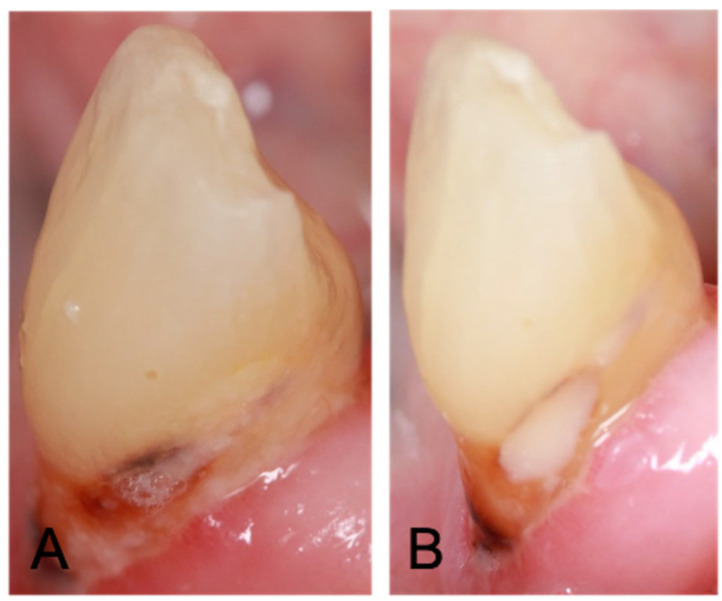
(**A**) Root caries lesion; (**B**) root caries lesion restored with GIC-CHX using the ART technique.

The other carious lesion (control tooth) was restored using conventional GIC (ChemFil Superior, DENTSPLY) with a flat plastic instrument or ball burnisher. Proprietary varnish (Chemfil Varnish™, Dentsply, Charlotte, NC, USA) was applied to the surface of the restoration. Excess material was removed with a carver. A representative restored root caries lesion is shown in Figure 1.

At baseline and 6 months after restoration placement, the survival, presence of marginal defects, and wear of the restorations were assessed by two independent operators. Clinical evaluation and assessment forms were based on the modified criteria proposed by Ryge [23] to evaluate the integrity of the restorations, anatomic form, the presence of recurrent caries, marginal adaptation, surface roughness, colour-match, and gingival health.

To assess the patient acceptability of the treatment, participants were given a questionnaire at baseline and at 6 months post-restoration. The questions were based on the smoothness of the restoration; pain experienced during and after the treatment, satisfaction with the aesthetics; changes in taste; anxiety and discomfort experienced during the procedure. The operator acceptability of the restorations was also assessed using a questionnaire.

### 2.3. Microbiological Analysis

**Figure 2:**
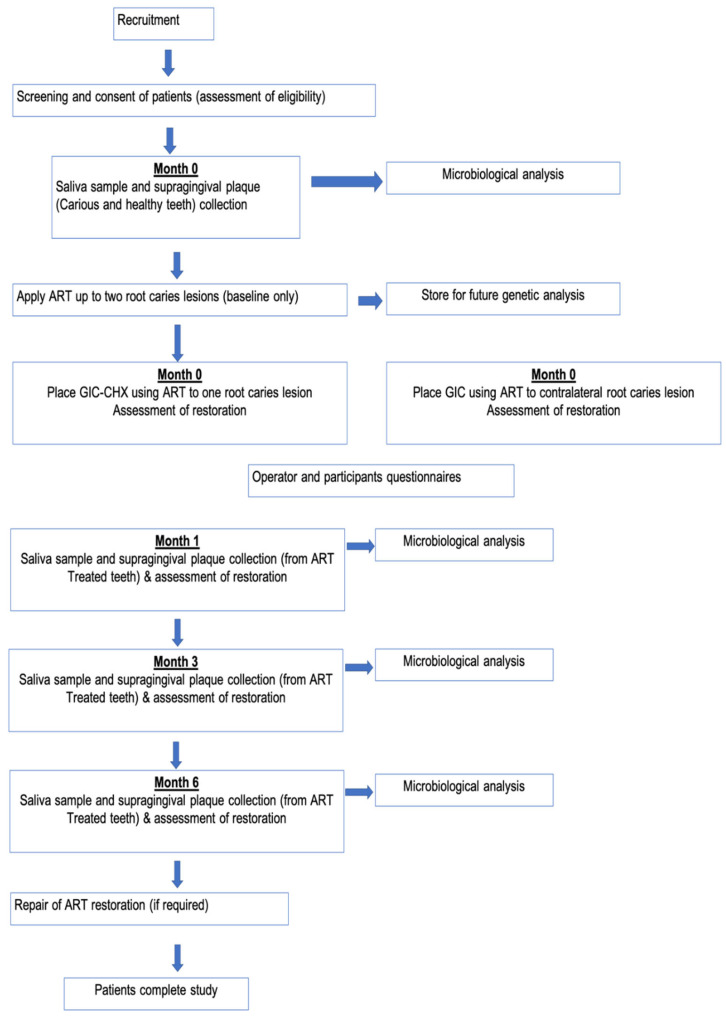
Flow chart for the microbiological study.

Plaque samples were collected before the placement of restorations for both test and control teeth. Saliva samples were also collected at baseline. All the samples were analysed for viable microorganisms. This procedure was repeated at 1, 3, and 6 months to test for the effect of GIC-CHX on the microbial counts. Figure 2 shows the steps involved in the microbiological part of the study.

#### Sample Collection

Prior to sampling, sterile 1.5 mL microfuge tubes were pre-labelled with a unique patient identification code, the corresponding stage of the study (0, 1, 3, or 6 months), the specimen type (GIC-CHX, GIC, or Saliva), and date of collection.

Each plaque sample was collected using a sterile interdental brush (TePe; TePe Munhygienprodukter AB, Malmö, Sweden). The head of the interdental brush was then cut with sterile scissors and placed into the labelled microfuge tube containing 1 mL of reduced transport fluid (RTF) (K_2_HPO_4_, KH_2_PO_4_, NaCl, (NH_4_)_2_SO_4_, Na_2_CO_3_, dithiothreitol, 0.1 M EDTA).

The microfuge tube containing RTF was weighed using a digital balance (ENTRIS623-1S, Sartorius Lab Instruments GmbH & Co. KG, Goettingen, Germany) before and after placement of the head of the interdental brush containing plaque samples, so that the weight of the plaque could be determined (*W_p_*; after subtracting the mean weight of brush heads). This procedure was conducted aseptically to reduce the possibility of contaminating the sample. Microfuge tubes were kept on ice during sample collection and transport.

Twelve 10-fold dilutions of oral samples were performed and 100 μl of each dilution was plated onto the following solid media (all supplied by Fort Richard Laboratories Ltd., Auckland, New Zealand): (i) Columbia sheep blood agar (CBA) as the non-selective medium for the enumeration of total viable microorganisms, (ii) mutans selective agar (MSA) for enumerating *Streptococcus mutans* and other cariogenic streptococci, (iii) Rogosa agar (RA) for cultivating *lactobacilli*, and (iv) Sabouraud dextrose agar (SDA) (with chloramphenicol) selective for *Candida* spp. and other yeasts. CBA, MSA, and RA plates were incubated anaerobically at 37 °C for 2 to 3 days; SDA was incubated at 30 °C aerobically for 3 to 5 days. Microbial counts from both test and control restorations were expressed as the number of colony-forming units (CFU) per milligram of plaque sample (CFU/mg) according to the following formula:

$$\frac{CFU}{mg}=\left(N_{c}\times DF\times V_{t}\right)/\left(V_{c}\times W_{p}\right)$$

where: 

*N_c_* = number of colonies counted for each plate;

*DF* = dilution factor;

*V_c_* = volume of culture plate (0.1 mL);

*V_t_* = total volume (1 mL);

*W_p_* = weight of plaque (mg).

### 2.4. Statistical Analysis

This is a pilot study, so formal power calculations were not appropriate. Numbers of patients recommended for pilot studies propose that the analysis dataset should comprise a minimum of 30 patients in order to estimate parameters for future sample size calculations. Similar studies using a similar population of patients have experienced a dropout rate of 10%. Therefore, the target sample size was increased to 34 to account for these. The actual power of the present study is communicated through providing 95% confidence intervals wherever possible. For the patient and operator outcomes, Wilcoxon signed rank tests were used for paired ordinals with four or more levels, tests of symmetry for paired ordinal data with three levels, and McNemar’s tests for paired binary data. All these tests were exact. For the microbiological analyses, the four CFU outcomes on each medium (SDA, CBA, MSA, and RA) were examined on the common logarithmic scale using four models in total, each incorporating the split mouth, and longitudinal data (with random effects for patients and patient-measurement occasion) for that outcome (up to 12 measurements/patient). There was no evidence against homogeneity of variances by time, treatment, or their combination based on Bayesian Information Criterion (BIC) values. The focus was on differences between unmodified GIC and GIC-CHX using changes from baseline to 1, 3, and 6 months post-restoration. Saliva was used as a reference/comparison. A time–location interaction was used to identify evidence of overall patterns of differences between the two treatments. Standard model diagnostics were performed.

All statistical analyses were performed using Stata 17 (StataCorp LLC, College Station, TX, USA.). Two-sided *p*-values < 0.05 were considered statistically significant. For the microbiological outcomes, the Holm–Bonferroni correction method was used to accommodate the four CFU outcomes when looking at individual timepoints. For the patient and operator outcomes, no adjustments were performed for the multiple comparisons.

## 3. Results

In total, 52 restorations were placed using both conventional GIC (control; *n* = 26) and GIC modified with chlorhexidine digluconate (test; GIC-CHX) (*n* = 26) in twenty-six participants. One patient, however, was lost to follow-up and they was only included in baseline patient responses and operator assessments.

### 3.1. Demographic Characteristics of Participants

Demographic details of the participants are summarised in Table 1.

### 3.2. Clinical Performance and Patient-Rated Acceptability of ART

At baseline, the majority of the participants (96%) were satisfied or very satisfied with the treatment, felt that the procedure was comfortable (92%), and did not feel pain (62%) (Table 2). At their 6-month review appointment, 84% of participants indicated that they were satisfied with the appearance of the restorations (Table 3). The operators found the ART treatment to be easy (81%) and quick (73%) to carry out (Table 4). At the 6-month examination, the operators were satisfied with the appearance and condition of both the GIC and GIC-CHX restorations, and differences between the types of restoration were not statistically significant (Table 5). A summary of patients’ and operators’ perceptions of the ART treatment at baseline and 6 months is shown in Table 3, Table 4 and Table 5.

At the 6-month examination, 48% (*n* = 17) of the test GIC-CHX restorations were still well adapted and two restorations had defective margins. In comparison, a higher proportion of the control GIC restorations had either catchy margins (*n* = 9; 36%) or obvious crevices (*n* = 10; 40%) (*p* = 0.036, exact test for symmetry) (Table 6). The surface roughness of the restorations was assessed according to the modified Ryge criteria. At 6 months, 60% of the GIC-CHX restorations were smooth, whereas many of the control GIC restorations were either slightly rough (*n* = 19; 76%) or rough (*n* = 2; 8%) (*p* = 0.002, exact test for symmetry). However, at baseline, more of the GIC-CHX restorations had a slight colour mismatch (*n* = 14; 56%) compared to control GIC restorations (*p* = 0.002). Two operators assessed the restorations at 6 months, and the survival of the restorations was based on the assessment of the blinded operator. The survival rates for GIC and GIC-CHX restorations were 80% and 92% respectively, and the main reason for failure was gross marginal defects. 

### 3.3. Microbiological Analysis

**Figure 3:**
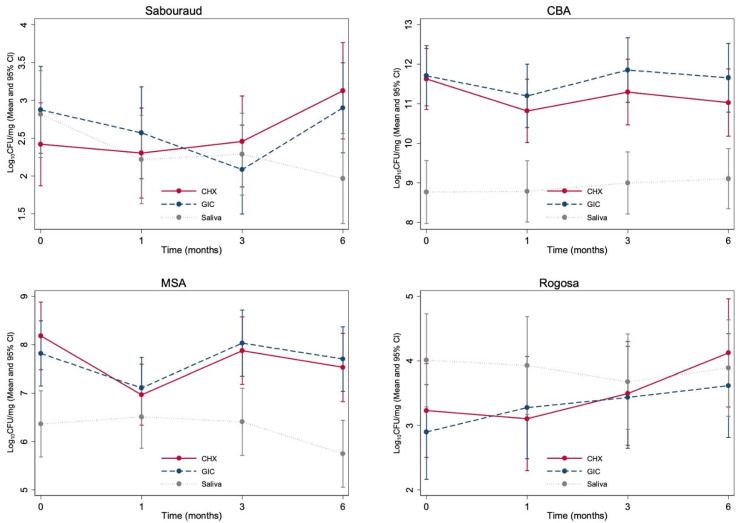
The mean counts of *Candida* (Sabouraud/SDA), total anaerobic bacteria (CBA), *Streptococcus mutans* (MSA), and lactobacilli (Rogosa) (Log_10_ (CFU/mg)) for plaque taken from GIC-CHX and control GIC restorations at each time period investigated and saliva (Log_10_ (CFU/mg). (Red: CHX; blue dashes: GIC; grey: saliva).

Figure 3 shows the mean counts of the microorganisms for plaque samples taken from around the cervical margins and interproximal areas of control GIC and test GIC-CHX restorations. Saliva samples were also collected for microbiological analysis, to measure the general microbiological effect of GIC-CHX in the oral cavity (Figure 3). Although the mean counts for all tested microorganisms were slightly reduced in plaque samples collected around the GIC-CHX restorations, after 6 months, the mean counts returned to baseline levels or higher. There was no evidence of differences between GIC and GIC-CHX in terms of changes over time. No time: treatment interactions were significant, nor were time-specific comparisons after accounting for multiple comparisons. Prior to adjustment for multiplicity, the only statistically significant result was the change from baseline to 3 months for *Candida* (SDA plates) (difference on the common log scale of 0.83, which translates to 6.8 times on the original scale, 95% CI 0.04–1.61, *p* = 0.038).

## 4. Discussion

This study assessed the clinical effectiveness, restoration integrity, survival, patient acceptability, and microbial colonisation of a modified glass ionomer cement restoration placed using ART to treat root caries. The survival rate of the modified GIC-CHX restorations was higher than for conventional GIC restorations over the 6-month period.

Most participants in this study found the procedure to be quick, comfortable, and minimally painful or uncomfortable. This is in agreement with several previous studies that have reported that patients experienced less pain during the placement of an ART restoration than with conventional restorative techniques [24,25]. Furthermore, the ART approach has other benefits, such as requiring only hand instruments to treat root caries, and the procedure can be carried out at rest homes where the elderly do not have to travel. Therefore, it provides a simple and cost-effective treatment modality which is a benefit to patients.

The cost-effectiveness of ART versus conventional restorative methods was assessed in a randomised clinical trial involving 82 adult patients in Ireland [15]. The study found that ART was more cost-effective than placing conventional restorations, with cost effectiveness ratios of 0.18 and 0.29, respectively. In addition, when a dental hygienist provided ART, the cost-effective ratio reduced to 0.14 [15], although this is not possible in some parts of the world because of the specific scope of practice for oral health therapists [26,27].

In this study, only 8% of the participants were anxious during the restorative procedure, and at the 6-month appointment, none of the participants were anxious. This high level of patient acceptability could be due to the participants being made aware that this simple procedure involved only hand instruments with no drilling or local anaesthesia. Despite two participants being anxious at the first visit, both reported not being anxious at subsequent visits, most likely because of favourable experiences during their first visits. This makes ART, where appropriate, a suitable treatment modality for dentally anxious patients, especially when a proper explanation of the procedure is delivered to the patient before treatment. A study conducted in South Africa [28] tested the hypothesis that ART would result in less dental anxiety compared to conventional restorations in outpatients attending public oral health clinics. The study found that ART caused less dental anxiety in both adults and children [28]. Participant satisfaction with the ART was also investigated, and all participants were satisfied with ART at baseline and at all subsequent appointments. A previous study conducted in Zimbabwe showed that 95% of secondary school students who had never previously received dental restorations were satisfied with the ART procedure and restorations [29]. Small and medium size cavities were found to be very easy to restore with ART by the operator; however, large cavities required extensive excavation, which may cause operating hand fatigue, so that the procedure takes longer than the conventional method which uses rotary instruments.

There was no change in taste perceived by participants with the GIC-CHX restorations. This indicates that the small amount of chlorhexidine in one GIC-CHX restoration may not have a significant effect on taste, even though chlorhexidine is known to impair taste when used as a mouthwash [30]. The setting times of the GIC-CHX restorations were similar to or quicker than those for control GIC restorations. This indicates that modification with chlorhexidine did not affect the setting time of GIC. A previous study found that altering the powder-to-liquid ratio affects the setting time, and a high powder-to-liquid ratio has been found to shorten the setting time of GIC [31].

In the present study, the clinical performance of GIC-CHX restorations was compared to that of control GIC restorations at baseline and 6 months using the modified Ryge criteria [23]. In addition to the operator who placed the restoration, another independent operator assessed the clinical performance of the restorations at six months to reduce operator-based bias. At the 6-month examination, marginal adaptation was assessed. Of the 25 restorations, 23 GIC-CHX restorations were either continuous or slightly catchy with no obvious crevice at the margin, whereas 10 control GIC restorations had obvious crevices at the margin. An obvious crevice at the margin is considered a failure according to the modified Ryge criteria [23]. Furthermore, no secondary caries lesions were evident in the test GIC-CHX restorations after 6 months (Table 6). A possible reason for this difference could be that GIC-CHX restorations eliminated the bacteria remaining in the cavity, preventing the development of secondary caries. A study by Lo et al. (2006) revealed that secondary caries was one of the main reasons for the failure of ART restorations when conventional GIC was used [25]. Secondary caries usually develops from the residual caries left in the prepared cavity, since the ART may not completely remove all the carious tissue [25]. Although fluoride-containing restorations such as GIC are known to have a cariostatic effect, it is not known whether the level of fluoride release is sufficient for inhibiting demineralisation [32]. However, incorporating an antibacterial agent in GIC may eliminate the remaining bacteria in the cavity, preventing secondary caries. De Castilho et al. (2013) showed that incorporation of chlorhexidine in resin-modified GIC eliminated all bacteria in the cavity when tested by re-entry into the cavity after 3 months [33].

Surface roughness was also assessed for both types of restorations. At 6 months, 60% of GIC-CHX restorations were smooth, whereas 84% of control GIC restorations were slightly rough or rough. This could have been due to faster wear of modified GIC-CHX in comparison to GIC. Marti et al. (2014) showed that the hardness of GIC decreased when chlorhexidine was added, which resulted in accelerated wear of the material [34].

In this study, the microbial counts in plaque samples from GIC-CHX restorations were not significantly lower than those around GIC restorations at all time points investigated. Previous studies have shown that incorporation of chlorhexidine in restorative materials increased the antibacterial effect of the material for up to 90 days [20,35,36]; however, these were in vitro studies, which did not replicate the oral environment, where saliva is produced and secreted continuously [37]. Given the fact that only a small amount of CHX was added to GIC, the high clearance rate of saliva could have reduced its effect significantly over time. In addition, fluid intake might have diluted the effect of chlorhexidine on the surface of the GIC and reduced its antimicrobial effect [38].

The slight reduction in cariogenic bacteria after 1 month could have been due the improvement of participants’ oral hygiene habits, following the instructions given at the time of the initial consultation. This may have reduced the plaque accumulation and number of cariogenic bacteria. Another possible reason for the reduction in cariogenic bacteria could be related to the elimination of carious dentine during the ART procedure, resulting in a change in the environment that was harbouring these bacteria prior to treatment. Furthermore, the potential antibacterial effect of fluoride release from GIC restorations should not be ignored [39], although some studies have suggested that the concentration of fluoride released is not high enough to result in significant antibacterial effects in GIC restorations [19]. Overall, it appears that the concentration of chlorhexidine incorporated in GIC was insufficient to reduce the numbers of the tested microorganisms.

This study has some limitations, including the limited number of restorations placed and the short period of follow-up. The recruitment of participants with two root caries lesions in a limited demographic region proved more difficult than originally anticipated. Other limitations, such as patients’ oral hygiene routine, general health conditions compromising the oral environment, and the size and location of placement of the restorations were accounted for as much as possible, but could have influenced the results to a certain extent. Despite this, the findings reported here are encouraging, and further clinical research is needed with a larger number of participants and a longer monitoring period. The optimum concentration of CHX that can be added to the GIC (without compromising restoration integrity) should also be investigated further.

## 5. Conclusions

Within the limitations of this study, placement of a CHX-modified GIC using the ART appears to provide a simple and minimally invasive restoration technique with better clinical outcomes which can be used to restore root caries. Although not statistically significant after accounting for multiple comparisons, GIC-CHX had better antibacterial outcomes at 1 month and resulted in higher restoration survival rates than unmodified GIC.

## Figures and Tables

**Table 1:** Summary of the patients’ demographic characteristics (*n* = 26).

	*n*	%
**Age**		
<30	2	8
50–59	6	23
60–69	10	38
>70	8	31
**Gender**		
Male	16	62
Female	10	38
**Ethnicity**		
New Zealand European	18	69
Māori	3	12
Asian	1	4
Other	4	15
**Smoking status**		
Former smoker	11	42
Never smoked	11	42
Current smoker	4	15
**Frequency of toothbrushing**		
Irregular	2	8
Regular: once a day	11	42
Regular: twice a day	13	50

**Table 2:** Patients’ perceptions of the ART technique at baseline (*n* = 26).

	*n*	%
**How comfortable was the ART?**		
Very comfortable	14	54
Comfortable	10	38
Neutral	0	0
Uncomfortable	2	8
Very Uncomfortable	0	0
**How much pain did you feel during the ART?**		
None	16	62
A little pain	10	38
A lot of pain	0	0
**How anxious did you feel during the ART?**		
Not at all anxious	24	92
A little anxious	2	8
Very anxious	0	0
**How anxious did you feel during your clinical visit?**		
Not at all anxious	21	81
A little anxious	5	19
Very anxious	0	0
**How much time did you feel that the ART procedure took?**		
Less than expected	2	8
As expected	11	42
More than expected	13	50
**How confident were you that the dentist was able to apply the treatment?**		
Very confident	19	73
Confident	7	27
Neutral	0	0
Unconfident	0	0
Very unconfident	0	0
**How satisfied are you with the appearance of the ART treatment of your tooth?**		
Very satisfied	10	38
Satisfied	15	58
Neutral	1	4
Dissatisdfied	0	0

**Table 3:** Patients’ perceptions of the ART treatment after 6 months.

	*n*	%
**How smooth does your treated tooth feel compared to your other teeth?**		
Very smooth	7	28
Quite smooth	9	36
Neutral	7	28
A little rough	2	8
Very rough	0	0
**How much pain did you have in your tooth/teeth following ART treatment?**		
None	23	92
A little pain	2	8
A lot of pain	0	0
**How anxious did you feel throughout your clinical visit?**		
Not at all anxious	25	100
A little anxious	0	0
Very anxious	0	0
**How satisfied are you with the appearance of the ART treatment on your tooth/teeth?**		
Very satisfied	11	44
Satisfied	10	40
Neutral	4	16
Unsatisfied	0	0
Very unsatisfied	0	0
**Have you experienced any taste change since receiving treatment?**		
None	24	96
A little change	1	4
A lot of change	0	0

**Table 4:** Clinical operators’ assessments at baseline (*n* = 26).

	*n*	%
**How easy was the ART procedure?**		
Very easy	9	35
Quite easy	12	46
Neutral	4	15
Quite difficult	1	4
Very difficult	0	0
**How long did the procedure take?**		
Less than expected	5	19
As expected	14	54
More than expected	7	27
**How satisfied are you with the appearance of the restoration following the ART treatment?**		
Very satisfied	7	27
Satisfied	14	54
Neutral	4	15
Unsatisfied	1	4
Very unsatisfied	0	0
**Time taken for GIC-CHX to set**		
Quicker than GIC	8	31
Same as GIC	14	54
Longer than GIC	4	15

**Table 5:** Clinical operators’ assessments at 6 months (*n* = 25).

How satisfied are you with the appearance of the patient’s tooth?					*p*-value
	GIC		GIC-CHX		0.995 *
	***n***	**%**	***n***	**%**
Very satisfied	4	16	4	16	
Satisfied	13	52	13	52	
Neutral	5	20	7	28	
Unsatisfied	1	4	0	0	
Very unsatisfied	2	8	1	4	
**How satisfied are you with the condition of the patient’s tooth?**					***p*** **-value**
	**GIC**		**GIC-CHX**		0.899 *
	***n***	**%**	***n***	**%**	
Very satisfied	3	12	3	12	
Satisfied	16	64	16	64	
Neutral	4	16	3	12	
Unsatisfied	0	0	2	8	
Very unsatisfied	2	8	1	1	

* Signed Wilcoxon test (exact *p*-value).

**Table 6:** Operator’s assessment using the modified Ryge criteria (*n* = 25).

1st Operator											2nd Operator				
	Baseline				*p*-Value	6 Months				*p*-Value	6 Months				*p*-Value
	GIC		GIC-CHX			GIC		GIC CHX			GIC		GIC CHX		
Anatomic form	*n*	%	*n*	%		*n*	%	*n*	%		*n*	%	*n*	%	
The restoration is continuous with the existing anatomic form	24	96	25	100	1 *	16	64	17	68	1 *	19	76	22	88	0.453
Slightly under/over contoured	1	4	0	0		9	36	8	32		6	24	3	12	
**Secondary caries**															
No visible evidence	25	100	25	100	1 *	23	92	25	100	0.5 *	25	100	25	100	1
Visible evidence	0	0	0	0		2	8	0	0		0	0	0	0	
**Marginal adaptation**															
Continuous with existing anatomic form	23	92	23	92	1 **	6	24	12	48	0.036 **	7	28	14	56	0.098
Explorer catches but no crevice visible	2	8	2	8		9	36	11	44		13	52	9	36	
Obvious crevice at margin, dentine or lute exposed	0	0		0		10	40	2	8		5	20	2	8	
**Surface roughness**															
Smooth	21	84	20	80	1 **	4	16	15	60	0.002 **	14	56	15	60	1
Slightly rough	4	16	5	20		19	76	10	40		11	44	10	40	
Rough	0	0	0	0		2	8	0	0		0	0	0	0	
**Colour match**															
Very good/good almost invisible	20	80	10	40	0.002 **	16	64	21	84	0.227 **	25	100	21	86	0.125
Slightly mismatch	5	20	14	56		9	36	4	16		0	0	4	14	
Gross mismatch outside of normal range	0	0	1	4		0	0	0	0		0	0	0	0	
**Gingival health**															
Healthy gingivae	13	52	5	20	0.112 ***	7	28	16	64	0.006 ***	0	0	0	0	**0.022**
Mild inflammation	6	24	12	48		13	52	9	36		8	32	15	60	
Moderate inflammation	5	20	8	32		5	20	0	0		15	60	10	40	
Severe inflammation	1	4	0	0		0	0	0	0		2	8	0	0	

* Exact McNemar test. ** Exact symmetry test. *** Exact Wilcoxon signed rank test.

## Data Availability

The data presented in this study are available on request from the corresponding author.
